# Mutational Analysis of a Red Fluorescent Protein-Based Calcium Ion Indicator

**DOI:** 10.3390/s130911507

**Published:** 2013-09-02

**Authors:** Haley J. Carlson, Robert E. Campbell

**Affiliations:** Department of Chemistry, University of Alberta, Edmonton, AB T6G 2G2, Canada; E-Mail: hcarlson@ualberta.ca

**Keywords:** red fluorescent protein, calcium ion, genetically encoded sensor, mutagenesis, spectroscopy

## Abstract

As part of an ongoing effort to develop genetically encoded calcium ion (Ca^2+^) indicators we recently described a new variant, designated CH-GECO2.1, that is a genetic chimera of the red fluorescent protein (FP) mCherry, calmodulin (CaM), and a peptide that binds to Ca^2+^-bound CaM. In contrast to the closely related Ca^2+^ indicator R-GECO1, CH-GECO2.1 is characterized by a much higher affinity for Ca^2+^ and a sensing mechanism that does not involve direct modulation of the chromophore p*K*_a_. To probe the structural basis underlying the differences between CH-GECO2.1 and R-GECO1, and to gain a better understanding of the mechanism of CH-GECO2.1, we have constructed, purified, and characterized a large number of variants with strategic amino acid substitutions. This effort led us to identify Gln163 as the key residue involved in the conformational change that transduces the Ca^2+^ binding event into a change in the chromophore environment. In addition, we demonstrate that many of the substitutions that differentiate CH-GECO2.1 and R-GECO1 have little influence on both the *K*_d_ for Ca^2+^ and the sensing mechanism, and that the interdomain linkers and interfaces play important roles.

## 1. Introduction

Molecular sensors that enable non-invasive fluorescence imaging of intracellular Ca^2+^ dynamics with high spatial and temporal resolution are powerful tools in modern cell biology and neuroscience research. As the Ca^2+^ ion is a universal “second messenger”, detection of elevated Ca^2+^ levels can reveal when an intracellular signaling pathway is being activated [1], or when an excitable cell is experiencing an action potential [2]. While organic dye-based Ca^2+^ indicators have long been a mainstay of such research [3], over the last decade proteinaceous Ca^2+^ indicators have emerged as a preferred alternative for many applications. The major advantage of proteinaceous Ca^2+^ indicators is that they are genetically encodable and thus can be tissue-selectively expressed and imaged in transgenic model organisms [4].

**Figure 1.**
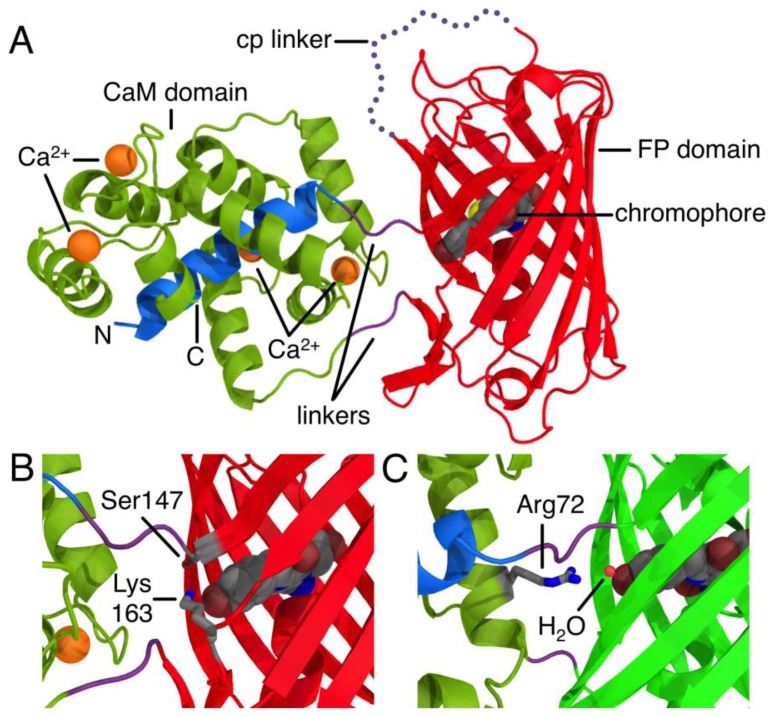
The structure of a single FP-based Ca^2+^ indicator. (**A**) Cartoon representation of the X-ray crystal structure of R-GECO1 [8]. Domains and linkers are colored according to the sequence alignment shown in Figure 2. The “cp linker” is the linker used to connect the original N- and C-termini of the circularly permuted (cp) FP. (**B**) Zoom in on the circular permutation site of R-GECO1, where the chromophore is exposed to the interface between the CaM and FP domains. (**C**) The circular permutation site of GCaMP2 (PDB ID 3EVR) represented from a similar perspective to (B) [9].

The development of proteinaceous Ca^2+^ indicators became a possibility only after the discovery, cloning, and subsequent optimization of the *Aequorea victoria* green FP [5]. Due to its inherent ability to generate a chromophore through an autonomous series of post-translational modifications, the green FP uniquely provides a means of genetically encoding a fluorophore. The first FP-derived Ca^2+^ indicators were based on the Ca^2+^-dependent modulation of Förster Resonance Energy Transfer (FRET) efficiency between two FP variants with different hues [1]. Subsequent protein engineering efforts led to the development FP-derived Ca^2+^ indicators based on a single FP that exhibited an intensiometric response of Ca^2+^ [6,7]. Such single FP-derived indicators are composed of a circularly permuted FP variant with new termini in close proximity to the centrally located chromophore. Fused to one of these new termini is the Ca^2+^-binding protein CaM, and fused to the other termini is the M13 peptide that binds to Ca^2+^-bound CaM (Figure 1A). The generally accepted mechanism of such sensors is that the chromophore is exposed to the solvent and the fluorescence is largely quenched in the absence of Ca^2+^. Binding of Ca^2+^ causes an interaction between CaM and its peptide binding partner that stabilizes the chromophore in a conformation and environment where fluorescent brightness is increased due to higher quantum yield and/or extinction coefficient. Such a stabilizing interaction is apparent in the X-ray crystal structure of R-GECO1, where a lysine side chain from a neighboring β-strand is stabilizing the fluorescent phenolate form of the chromophore in the Ca^2+^-bound state (Figure 1B) [8]. Similarly, in GCaMP2 [9,10], an arginine side chain from CaM bridges the CaM to FP interface and stabilize the phenolate form of the chromophore in the Ca^2+^-bound state (Figure 1C).

Since 2001 when the single FP Ca^2+^ indicator design was first reported [6,7], dedicated optimization efforts have produced an ever-improving series of improved GCaMP variants [2,11–13]. Recent years have also seen the introduction of an expanded color palette of Ca^2+^ indicators based on both engineered versions of *Aequorea* green FP [8,14] as well as homologous red fluorescent Anthozoan FPs from organisms such as *Entacmaea quadricolor* sea anemone [8] and *Discosoma sp.* coral [14,15]. The prototypical red fluorescent indicator is R-GECO1 [14] that was engineered from the *Discosoma*-derived FP known as mApple [16]. Some of the most recent additions to this growing selection of red fluorescent indicators are CH-GECO2.0 and CH-GECO2.1 [17], which were engineered from the popular *Discosoma*-derived red FP known as mCherry [18].

**Figure 2.**
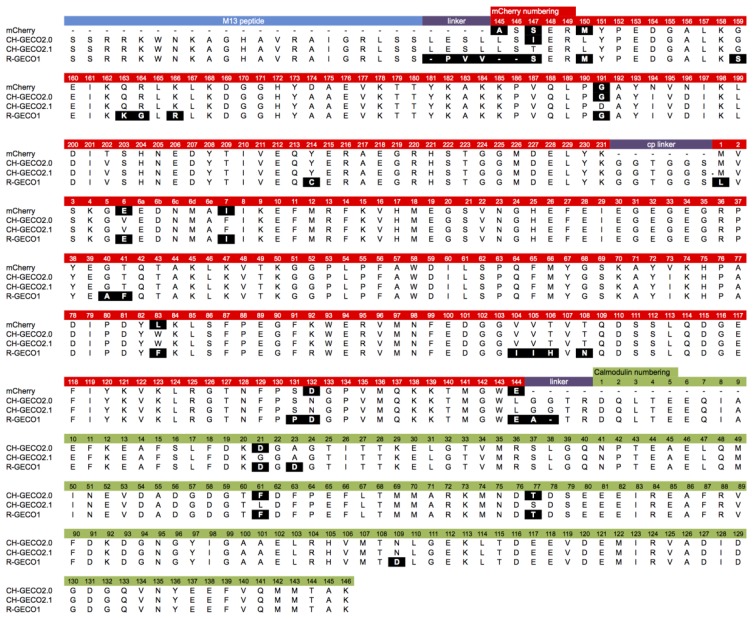
Sequence alignment of Ca^2+^ indicators discussed in this work. Substitutions relative to CH-GECO2.1 are highlighted with white text on a black background. Adapted in part from Carlson and Campbell [17].

Although they are both ultimately derived from the same wild-type protein, the extensive processes of directed evolution that led to R-GECO1 and CH-GECO2.1 have introduced a considerable number of differences in the amino acid sequences (Figure 2). These differences include 26 amino acid substitutions (Leu1Met, Glu6Val, Ile7Phe, Ala40Gly, Phe41Thr, Phe83Trp, Ile104Val, Ile105Val, His106Thr, Asn108Thr, Pro131Ser, Asp132Asn, Glu144Leu, Ser147Thr, Met150Leu, Ser159Gly, Lys163Gln, Gly164Arg, Arg166Lys, Gly191Asp, Cys214Tyr, Asp21Gly (CaM), Asp23Ala (CaM), Phe61Leu (CaM), Thr77Ser (CaM), and Asp109Asn (CaM), relative to R-GECO1), as well as differences in the interdomain linkers. In addition, they exhibit some quite dramatic differences in their respective *K_d_*s for binding to Ca^2+^ (6 nM for CH-GECO2.1 *vs.* 480 nM for R-GECO1) and even in the underlying mechanism by which this sensing occurs [17]. Specifically, R-GECO1 operates on the basis of Ca^2+^-dependent shift in the chromophore p*K*_a_ [14], whereas the Ca^2+^ sensing mechanism of CH-GECO2.1 appears to depend on the interaction of the chromophore with a yet unidentified ionizable amino acid side chain with a p*K*_a_ of ∼6.5 to 7.0 [17].

In an effort to identify which amino acid substitutions are responsible for the differences between CH-GECO2.1 and R-GECO1, and to obtain insight into the mechanism of CH-GECO2.1, we now report the characterization of a barrage of single-site mutants of CH-GECO2.1. Each variant has been characterized in terms of its Ca^2+^*K*_d_ as well as its fluorescence intensity as a function of pH both in the presence and absence of Ca^2+^. Interpreting the results of these studies in the context of the mCherry and R-GECO1 crystal structures has allowed us to propose a mechanistic basis for the response of CH-GECO2.1 to Ca^2+^ binding.

## 2. Materials and Methods

### 2.1. Mutagenesis

All molecular biology procedures were carried out using genes encoding either CH-GECO2.0, CH-GECO2.1, or R-GECO1 in pBAD/His B, as previously described [17]. All site-directed mutagenesis was performed using the Quikchange lightning mutagenesis kit (Agilent, Santa Clara, CA, USA) and primers designed according to the manufacturers guidelines.

### 2.2. Plasmid Purification

All plasmid DNA was purified from bacteria using a chloroform extraction protocol. Briefly, 150 μL of solution I (50 mM Tris, 10 mM EDTA, 100 μg/mL RNaseA, pH 8.0) was used to resuspend the bacterial pellet. Then 150 μL of solution II (1% SDS, 0.2 M NaOH) was added and the mixture is gently inverted several times. Solution III (2 M acetic acid, 3 M KOAc, pH 5.5) was added to a total volume of 450 μL and mixed to pellet the non-soluble cell debris. Finally, 150 μL of chloroform was added and mixed several times before being centrifuged at 14,000 rpm, 4 °C for 5 min. The top aqueous layer was separated, mixed with 800 μL of 100% ethanol, and then centrifuged at 4 °C for 5 min. The DNA pellet was washed with 500 μL of 70% ethanol, allowed to air-dry, and then dissolved in distilled water. Coding sequences of all gene variants were sequenced using BigDye Terminator v3.1 Cycle Sequencing Kit (Life Technologies, Carlsbad, CA, USA) and reactions were analyzed by the University of Alberta Molecular Biology Services Unit.

### 2.3. Protein Expression and Purification

*Escherichia coli* strain DH10B was transformed with the plasmid of interest by electroporation. Transformed bacteria were grown overnight on solid media containing ampicillin, and then a single colony was picked and grown overnight in 5 mL Luria broth (LB) supplemented with ampicillin at 37 °C. The 5 mL culture was then used to inoculate 250 mL of Terrific broth (TB) and grown to an optical density of 0.6. Protein expression was induced with 0.004% arabinose and the culture was grown overnight at 37 °C or for two nights at 30 °C, depending on the brightness of the protein construct. Bacteria were pelleted at 10,000 rpm, 4 °C for 10 min and the pellet was then resuspended in 10 mM Tris-Cl, 150 mM NaCl pH 7.4 at 4 °C. Cells were lysed using a cell disruptor (Constant Systems Ltd., Daventry, United Kingdom) and the debris pelleted at 14,000 rpm. Protein was purified from the supernatant by Ni-NTA affinity chromatography (Qiagen, Hilden, Germany) according to the manufactures instructions. Briefly, Ni-NTA beads were collected on a column with a vacuum manifold and washed twice with 10 mM Tris-Cl, 30 mM imidazole, 150 mM NaCl pH 8.0. The beads were gravity washed once and then eluted with 300 mM imidazole, 10 mM Tris-Cl pH 8.0. The excess imidazole was removed via buffer exchange with Amicon columns (MWCO 10,000) and 10 mM Tris-Cl, 150 mM NaCl, pH 7.3.

### 2.4. pH Titrations

Fluorescence intensity as a function of pH was determined by dispensing 5 μL of the protein solution into 50 μL of the desired pH buffer in triplicate into a 396-well clear-bottomed plate (Thermo Fisher Scientific, Waltham, MA, USA). Buffer solutions were prepared by adjusting the pH of a solution of 30 mM trisodium citrate and 30 mM sodium borate to pH 11.5. The pH of the solution was then adjusted with HCl (12 M and 1 M) and 10–15 mL was collected at pH value intervals of 0.5, plus two additional solutions at pH 5.25 and 5.75. Buffers were prepared both without (30 mM MOPS, 100 mM KCl, 10 mM EGTA, pH 7.2) and with (30 mM MOPS, 100 mM KCl, 10 mM CaEGTA, pH 7.2) Ca^2+^. Fluorescence emission for each solution was recorded using a Tecan (Maennedorf, Switzerland) Safire2 microplate reader.

### 2.5. Ca^2+^ Titrations

The apparent *K*_d_ for Ca^2+^ response was determined by mixing the protein solution with buffers containing various amount of Ca^2+^, prepared as described in the Calcium Calibration Buffer Kit from Life Technologies (Carlsbad, CA, USA). The Ca^2+^-free buffer (30 mM MOPS, 100 mM KCl, 10 mM EGTA, pH 7.2) and Ca^2+^-saturated buffer (30 mM MOPS, 100 mM KCl, 10 mM CaEGTA, pH 7.2) were mixed in different ratios to generate buffers with Ca^2+^ concentrations ranging from zero Ca^2+^ to 39 μM Ca^2+^. Similar to the pH titrations, 5–10 μL of the protein was mixed with 150–200 μL of each Ca^2+^ buffer. 50 μL of each solution was aliquotted in triplicate into a 396-well plate and the fluorescence emission was recorded using the plate reader. Emission peaks were integrated and plotted against the log of the calculated free Ca^2+^ concentration. Ca^2+^ titration curves were fit with a sigmoidal curve in order to obtain the *K*_d_ and the Hill coefficient.

## 3. Results and Discussion

### 3.1. Probing the Determinants of the Ca^2+^-Binding Affinity

**Figure 3.**
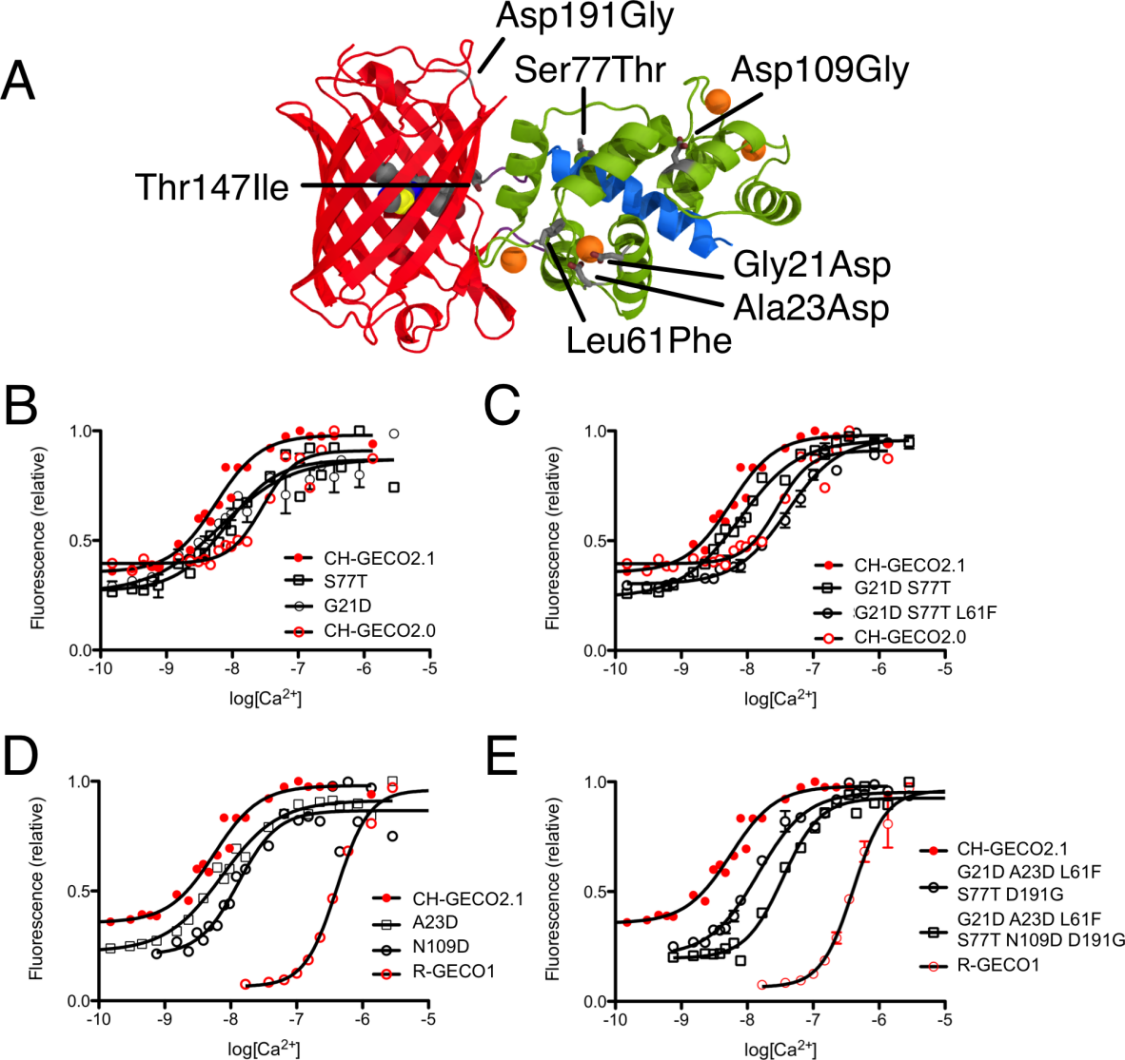
Probing the differences in Ca^2+^ affinity between CH-GECO2.0, CH-GECO2.1, and R-GECO1. (**A**) Location of amino acid substitutions that differentiate the indicators, represented using the R-GECO1 crystal structure [8]. Mutations are labeled relative to CH-GECO2.1. (**B**) Ca^2+^ titration curves for variants with single mutations that revert CH-GECO2.1 CaM to CH-GECO2.0 CaM. (**C**) Ca^2+^ titration curves for variants with multiple mutations that revert the CaM domain of CH-GECO2.1 to that of CH-GECO2.0. (**D**) Ca^2+^ titrations for two of the point mutants that revert CH-GECO2.1 to R-GECO1. (**E**) Ca^2+^ titrations for CH-GECO2.1 variants with the CaM domain partially and completely converted to the R-GECO1 CaM domain.

CH-GECO2.0 (*K*_d_ = 28 nM) and CH-GECO2.1 (*K*_d_ = 6 nM) [17] differ by only 3 substitutions in the CaM domain (Gly21Asp, Leu61Phe, and Ser77Thr, relative to CH-GECO2.1) and 2 substitutions in the FP domain (Thr147Ile and Asp191Gly) (Figures 2 and 3A). However the *K*_d_s for Ca^2+^ of these two proteins differ by a factor of 4.7. Furthermore, CH-GECO2.0 and R-GECO1 (*K*_d_ = 480 nM) [14] differ by only 1 substitution in the CaM domain (Ala23Asp, relative to CH-GECO2.0), but have *K*_d_s that differ by a factor of 17. The M13 peptide domain of all three proteins is identical. To determine which individual mutations, or combination of mutations, were responsible for the differences in *K*_d_ values, we used site directed mutagenesis to systematically revert mutations and then determined the effect on Ca^2+^ affinity. Notably, in wild-type CaM, Asp21 and Asp23 are two of the key Ca^2+^ chelating residues of the first EF hand [19].

We first introduced mutations to revert the CaM sequence of CH-GECO2.1 back to CH-GECO2.0. Results for all mutations discussed in this work are summarized in Supplementary Table 1. We determined that CH-GECO2.1 with Gly21Asp or Ser77Thr gave *K*_d_ values of 7 nM and 8 nM respectively (Figure 3B). We were unable to purify any soluble protein for CH-GECO2.1 Leu61Phe, which is located near the second EF hand of CaM. Previous studies of CaM have demonstrated that mutations in the second EF hand are more detrimental to the Ca^2+^ affinity than mutations in the first EF hand [20]. The combination of Gly21Asp and Ser77Thr results in a *K*_d_ of 13 nM, which is somewhat higher than either substitution alone. Addition of the Leu61Phe mutation yielded a *K*_d_ of 44 nM for the triple mutant (Figure 3C). Overall, this result demonstrates that these three mutations in the CaM domain account for most of the difference between the *K*_d_ values of CH-GECO2.0 and CH-GECO2.1. The fact that the *K*_d_ values of CH-GECO2.0 (28 nM) and the triple mutant of CH-GECO2.1 (44 nM) are not identical indicates that the two substitutions in the FP domain (Ile147Thr and Gly191Asp) must also have a subtle influence on the *K*_d_, likely through interactions at the interface between CaM and the FP domain.

We next introduced mutations intended to probe the differences between the CaM domain of R-GECO1 (*K*_d_ = 480 nM) and CH-GECO2.1 (*K*_d_ = 6 nM) variants, which have almost a 2-order of magnitude difference in their *K*_d_s for Ca^2+^. These two proteins differ by a total of 5 substitutions in the CaM domain (Gly21Asp, Ala23Asp, Leu61Phe, Ser77Thr, and Asn109Asp, relative to CH-GECO2.1) (Figures 2 and 3A). Introduction of the single mutations Ala23Asp or Asn109Asp into CH-GECO2.1 gave essentially unchanged *K*_d_s of 7 nM and 13 nM, respectively (Figure 3D). To fully convert the CH-GECO2.1 CaM to that of R-GECO1 CaM we proceeded to introduce additional substitutions. As discussed above, CH-GECO2.1 with Gly21Asp, Leu61Phe and Ser77Thr had an increased *K*_d_ of 44 nM. The addition of Ala23Asp slightly lowered the *K*_d_ to 23 nM. Further reversion of a mutation close to the FP-CaM interface (Asp191Gly) and one final mutation in CaM that is relatively distant from the EF hands (Asn109Asp) produced a protein with a *K*_d_ of 35 nM (Figure 3E). These results clearly demonstrate that the dramatic difference in *K*_d_ between R-GECO1 and CH-GECO2.1 cannot be fully explained by the mutations within the CaM domain. Rather, these changes must be due to the interactions between the CaM and FP domains, either via the linkers or the domain interface.

### 3.2. Investigating the Mechanism of CH-GECO Variants

To gain a better understanding of the differences in magnitude and mechanisms of the fluorescent responses of CH-GECO2.0, CH-GECO2.1, and R-GECO1, we created, characterized, and compared a series of protein variants with strategic substitutions at key residues of interest.

#### 3.2.1. CH-GECO2.0 *vs.* CH-GECO2.1

Just two mutations in the FP domain (Ile147Thr and Gly191Asp, relative to CH-GECO2.0) differentiate CH-GECO2.0 and CH-GECO2.1, but their fluorescent responses to Ca^2+^ are 150% and 250%, respectively. While the Asp191Gly reversion variant of CH-GECO2.1 variant retained a similar response to CH-GECO2.1, reversion of position 147 to Ile resulted in a decrease in the Ca^2+^ response to 115%. This result indicates that the Ile147Thr mutation is primarily responsible for the improved performance of CH-GECO2.1, and is consistent with the fact that the side chain of residue 147 is in close proximity to the phenolate moiety of the chromophore (Figure 1B).

#### 3.2.2. R-GECO1 *vs.* CH-GECO2.1: The Influence of the Linkers

Previous work in our lab with both single FP-based and FRET-based indicators has showed that the length and composition of the linkers between the protein domains can have a substantial effect on the response of the indicator to the analyte of interest [14,21,22]. The sequence alignment between R-GECO1 and CH-GECO2.1 (Figure 2) reveals that the linkers between the three domains are significantly different. Specifically, R-GECO1 has a shorter linker between M13 and the FP (Pro-Val-Val) than does CH-GECO2.1 (Leu-Glu-Ser-Leu) and the first two residues (Leu145 and Ser146) of the FP domain are deleted. R-GECO1 also has a shorter linker between CaM and the FP domain (Ala-Thr-Arg) than does CH-GECO2.1 (Gly-Gly-Thr-Arg). The Thr-Arg residues of both linkers correspond to the translation of a *MluI* restriction site.

**Figure 4.**
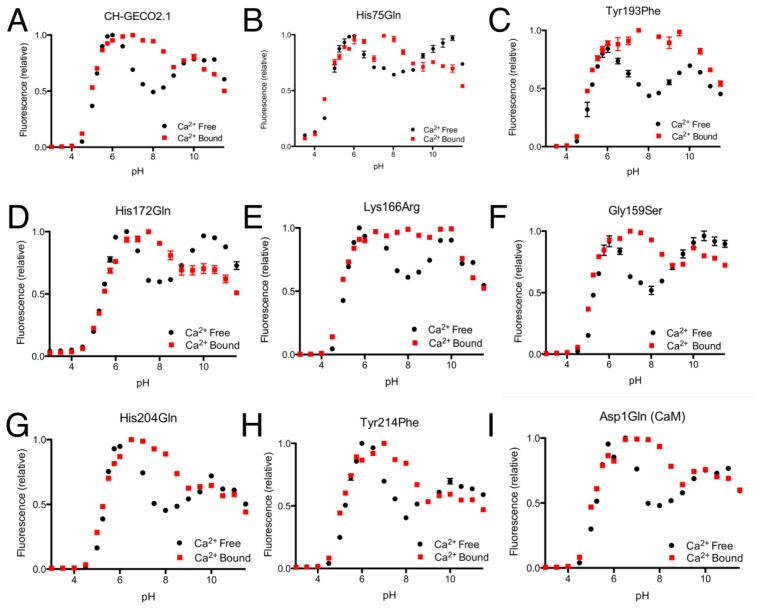
Representative fluorescence *vs.* pH titration curves for CH-GECO2.1 variants. (**A**) CH-GECO2.1 titration in the presence and absence of Ca^2+^, adapted from Carlson and Campbell [17]. (**B**–**I**) Titration curves for additional variants discussed in the text, both in the presence and absence of Ca^2+^.

Replacing the Leu-Glu-Ser-Leu linker and residues 145 and 146 of CH-GECO2.1 with Pro-Val-Val resulted in a dim red fluorescent protein (designated CH-GECO2.1-PVV). UV-vis spectroscopy revealed that a large portion of the protein was halted at the blue intermediate species that absorbs at 382 nm (Supplementary Figure 1A) [23]. As shown in Supplementary Figure 1C, the pH dependence of the red fluorescent component of CH-GECO2.1-PVV was quite different from that of CH-GECO2.1 (Figure 4A), and the response to Ca^2+^ was effectively abolished. Replacing the FP to CaM Gly-Gly-Thr-Arg linker of CH-GECO2.1 with Ala-Thr-Arg and introducing the adjacent Leu144Asp mutation, fully converted the CH-GECO2.1 into a variant with identical linkers to R-GECO1 (designated CH-GECO2.1-PVV-ATA). However, no red fluorescent protein could be recovered from repeated attempts to express this protein. These results indicate that, in addition to their critical role in the Ca^2+^ response mechanism of single FP indicators, the interdomain linkers can also have an important role in the chromophore maturation.

#### 3.2.3. Attempts to Identify Key Residues Involved in the Ca^2+^ Response of CH-GECO2.1

From the CH-GECO2.1 pH titration curve reproduced in Figure 4A, it is apparent that there is a key ionization with a p*K*_a_ around pH 6.5 to 7.0. We hypothesized that, at physiological pH and in the absence of Ca^2+^, there is a specific residue (of unknown identity) for which the deprotonated form is quenching the fluorescence of the chromophore [17]. In the presence of Ca^2+^ either this residue is no longer deprotonated due to a change in p*K*_a_, or it is no longer in the right conformation to quench the chromophore fluorescence. In an attempt to determine the identity of the residue that is so critical to the function of the CH-GECO variants, we performed mutagenesis of rationally selected candidate residues.

As the p*K*_a_ in question is around 6.5 to 7.0, the most likely candidate residue was histidine. However, the X-ray crystal structure of mCherry [24] reveals no histidines in the immediate vicinity of the chromophore, so we looked to histidine residues that could reasonably be interacting with the chromophore by an extended hydrogen bond network. One likely candidate was His75, which appears to participate in a hydrogen bond network with Tyr193, Lys70, Glu148, several water molecules, and the chromophore (Supplementary Figure 2A) in the mCherry crystal structure [24]. We mutated each of these residues individually and characterized the pH profile and *K*_d_ for the variants that formed the red fluorescent chromophore. No red fluorescent protein could be obtained for Lys70Gln and Glu148Gln, and absorbance scans of both variants revealed that the chromophore maturation process was stuck at the blue intermediate species similar to CH-GECO2.1-PVV described above [23]. Mutation His75Gln gave a pH profile that was very similar to CH-GECO2.1, albeit with a reduced response of 158% (Figure 4B). The Tyr193Phe mutation yielded similar results with a pH curve (Figure 4C) and Ca^2+^ affinity that are similar to CH-GECO2.1. We conclude that this hydrogen bond network is not involved in the Ca^2+^-dependent response of the CH-GECO variants, but does play an important role in the chromophore maturation process.

Having ruled out our original hydrogen-bond network hypothesis, we reexamined the mCherry crystal structure and identified Tyr214, His204, His172, and Asp1 of CaM as ionizable functional group in reasonably close proximity to the chromophore (Supplementary Figure 2B). Each of these residues was mutated to non-ionizable alternatives in the context of CH-GECO2.1, and the pH profiles both with and without Ca^2+^ were determined (Figure 4F–I). The pH profiles of all four mutants still retained the characteristic fluorescence increase at pH 6.5 to 7.0, indicating that none of these residues played a part in the Ca^2+^ sensing mechanism. Notably, Asp1 is the first amino acid of CaM and mutation to Gln did not alter the pH dependence, but it did increase the *K*_d_ to 53 nM and the fluorescence response to 307%. Due to its improved fluorescence response and a *K*_d_ that is very similar to that of the high affinity Ca^2+^ indictor YC-Nano50 [25], CH-GECO2.1 Asp1Gln may be particularly useful for imaging of transient changes in Ca^2+^ concentration in cells with low resting concentrations.

Expanding our efforts to include non-ionizable residues close to the chromophore, we decided to target positions 159, 163, and 166, which differ between R-GECO1 and CH-GECO2.1 (Supplementary Figure 2B). Introducing the Gly159Ser (Figure 4D) or Lys166Arg (Figure 4E) substitutions into CH-GECO2.1 resulted in variants where no substantial changes were observed in the *K*_d_ or excitation and emission characteristics, and only modest effect on the Ca^2+^ response in the case of the Gly159Ser variant (Supplementary Table 1). In contrast, the Gln163Lys substitution completely abolished the fluorescent response of CH-GECO2.1. This variant will be discussed in more detail in a following section. From these results it is apparent that, of all the residues targeted in these efforts, only Gln163 plays a critical role in the mechanism of action of CH-GECO2.1.

#### 3.2.4. The Influence of Residue Gln163 on the Ca^2+^ Response of CH-GECO2.1

Residue 163 of DsRed [26], and its engineered descendants such as mRFP1 [27] and mCherry [18], is in close proximity to the chromophore and thus substitutions at this position can have profound effects on the fluorescent characteristics. In wild-type DsRed, residue 163 is a Lys, which stabilizes the phenolate group of the chromophore in the anionic state by interaction with the protonated terminal amine [26]. During the directed evolution of dimeric and monomeric DsRed variants, favorable substitutions at this position included Lys163Gln in the dimer2 variant and Lys163Met in mRFP1 [27]. During later directed evolution of mRFP1 that ultimately led to the mCherry variant, the Met163Gln mutation was rediscovered and retained since this mutation provided improved chromophore maturation efficiency [18]. In contrast, the Met163Lys reversion was found to be beneficial during the directed evolution that led to the mApple variant [16]. Through the directed evolution process that led to CH-GECO2.1, the Lys163Gln substitution inherited from mCherry was retained. Notably, this residue is found in close proximity to both the chromophore and the circular permutation site, so it is prime candidate for a residue that would play a crucial role in the Ca^2+^ sensing mechanism.

The X-ray crystal structures of R-GECO1 and RCaMP (engineered from *Entacmaea quadricolor*-derived mRuby [28]) have recently been reported [8]. In the R-GECO1 structure, Lys163 is interacting directly with the phenolate of the chromophore in the Ca^2+^ bound state (Figure 1B), which indicates that this residue almost certainly has a critical role in the Ca^2+^ response, likely lowering the pK_a_ of the chromophore in the presence of Ca^2+^ by stabilizing the negative charge on the phenolate. The structurally equivalent residue in RCaMP [8] is a methionine that is directed inside the barrel in a conformation that is very similar to that of Lys163Gln in mCherry [24].

**Figure 5.**
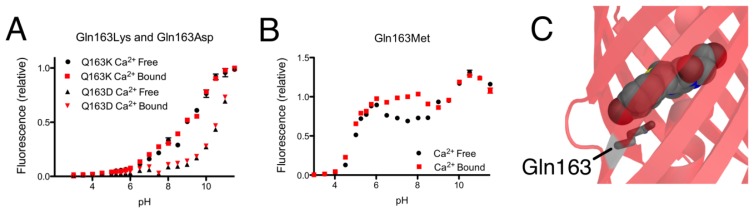
Effect of mutations at Gln163. (**A**) The Gln163Lys and Gln163Asp mutations abolish the response to Ca^2+^. (**B**) The Gln163Met mutant retains a response to Ca^2+^. (**C**) In the X-ray structure of mCherry (PDB ID 2H5Q) [24], Gln163 is directed into the interior of the protein in close proximity to the chromophore. Compare the orientation of Gln163 with the orientation of Lys163 in R-GECO1 as shown in Figure 1B.

As mentioned in the previous section, introducing the Gln163Lys substitution into CH-GECO2.1 completely abolished the response of CH-GECO2.1 and had a pronounced effect the protonation state of the chromophore as evidenced by the pH titration curve shown in Figure 5A. The absorbance, excitation, and emission wavelengths remain unchanged. To determine if the combination of Gln163Lys and R-GECO1-type linkers would be sufficient for rescuing the Ca^2+^ responsiveness, the Gln163Lys substitution was introduced into CH-GECO2.1-PVV-ATA. The resulting variant formed the red fluorescent chromophore but did not show a significant fluorescence response to Ca^2+^ (Supplementary Figure 1B,C).

To further probe the role of Gln163, we created the Gln163Met and Gln163Asp variants of CH-GECO2.1. Intriguingly, the Gln163Met variant behaved very similar to CH-GECO2.1 in terms of its response to Ca^2+^, though the Ca^2+^ response decreased to 54% and the *K*_d_ decreases to 2 nM (Figure 5B). Much like the Gln163Lys variant, the Gln163Asp variant exhibited no response to Ca^2+^ (Figure 5A). Based on these results, we concluded that Gln163 plays a critical role in the CH-GECO2.1 sensing mechanism, and that this role is distinct from that played by the equivalent Lys163 residue in R-GECO1.

#### 3.2.5. A Proposed Ca^2+^ Sensing Mechanism for CH-GECO2.1

The combined use of site directed mutagenesis and detailed *in vitro* characterization has enabled us to identify Gln163 as one of the key residues in the Ca^2+^ sensing mechanism of CH-GECO2.1. In the absence of a crystal structure of CH-GECO2.1, it is of course difficult to predict how specific amino acids are oriented near the chromophore, especially given that we expect substantial distortion of the protein (relative to mCherry [24]) in the vicinity of the circular permutation site. Fortunately, the X-ray structures of R-GECO1 and RCaMP are available to aid us in our effort to interpret our results.

We propose that, in the Ca^2+^ free state, the side chain of Gln163 is directed outside the barrel similar to the orientation of Lys163 in R-GECO1 (Figure 1B). In this orientation the chromophore is less sterically constrained by the surrounding protein environment and thus has more rotational and vibrational freedom, which explains the low quantum yield in the absence of Ca^2+^. When CaM and M13 interact due to Ca^2+^ binding, the side chain of Gln163 moves inwards and interacts with the chromophore in a conformation similar to that observed in mCherry (Figure 5C). That is, the side chain of Gln163 is likely oriented below the chromophore and sandwiched between Trp143 and Ile161. Presumably, this interaction leads to a stabilization of the chromophore in a planar conformation that increases the quantum yield. This proposed mechanism is consistent with the results obtained with the Gln163Met, Gln163Asp, and Gln163Lys variants. Mutation to the hydrophobic Met produces a functional protein, since the side chain is still able to undergo the Ca^2+^-dependent switch from the exterior to the interior of the protein. In contrast, mutation of Gln163 to either Lys or Asp dramatically increases the energetic cost of bringing the charged side chain into the hydrophobic interior, and the Ca^2+^ sensing mechanism is abolished.

Disappointingly, we were not able to unambiguously identify key residues the contributed to the much higher affinity for Ca^2+^ of CH-GECO2.1 relative to R-GECO1, nor we were able to identify the key residue that is undergoing an ionization with a p*K*_a_ ∼6.5 to 7.0. We suspect that, in both cases, the relevant residues are located in the interface between the CaM domain and the FP domain.

## 4. Conclusions

The goal of this work was to gain insight into the mechanistic details of the recently reported genetically encoded Ca^2+^ indicator CH-GECO2.1. While we were not able to answer all of the questions that naturally emerged from this effort, we were successful in identifying Gln163 as the most important residue for transducing the conformational change associated with Ca^2+^ binding into a change in the fluorescence intensity of the FP domain. We expect that this insight will be critical in guiding future efforts to further optimize genetically encoded Ca^2+^ indicators for improved response. Another important conclusion from this work is that interdomain connections and interactions can have a complex influence on multiple aspects of a genetically encoded biosensor. Specifically, we observed that changes in linker composition and length could effect protein maturation and the sensing mechanism. In addition, our results suggest that interfacial residues play a crucial role in determining the affinity of the indicator for Ca^2+^. We recommend that future efforts to optimize such indicators should always involve substantial screening of linker libraries that vary in both length and composition. Also, we suggest that, given the complexity of the sensing mechanisms, directed evolution efforts that involve screening of libraries of randomly generated variants will tend to be much more effective than efforts guided by naive mechanistic hypotheses.

## Supplementary Material
