# Supplementary material for: Mutational Analysis of a Red Fluorescent Protein-Based Calcium Ion Indicator
Source: Sensors (Basel). 2013 Sep 2;13(9):11507–21. doi: 10.3390/s130911507 (PMC3821290; doi:10.3390/s130911507)
Supplement: Supplementary file 1 [file sensors-13-11507-s001.pdf]

OPEN ACCESS

sensors

ISSN 1424-8220

www.mdpi.com/journal/sensors

Supplementary Information

Mutational Analysis of a Red Fluorescent Protein-Based Calcium Ion Indicator. *Sensors* 2013, *13*, 11507-11521

Haley J. Carlson and Robert E. Campbell *

Department of Chemistry, University of Alberta, Edmonton, AB T6G 2G2, Canada;
E-Mail: hcarlson@ualberta.ca

* Author to whom correspondence should be addressed; E-Mail: robert.e.campbell@ualberta.ca;
Tel.: +780-492-1849; Fax: +780-492-8231.

Supplementary Table 1. Characterization of CH-GECO2.0 and CH-GECO2.1 mutants.

Protein	Domain	Mutation	Response (%)	K_d (nM)	Hill Coefficient
CH-GECO2.0	N/A	N/A	150	28 ±2.4	1.7 ±0.2
CH-GECO2.1	N/A	N/A	250	6 ±0.3	1.3 ±0.1
CH-GECO2.1	CaM	Ser77Thr	218	8 ±1.1	1.1 ±0.1
CH-GECO2.1	CaM	Gly21Asp	260	7 ±1.4	0.7 ±0.1
CH-GECO2.1	CaM	Leu61Phe	no soluble protein		
CH-GECO2.1	CaM	Gly21Asp Ser77Thr	280	13 ±0.7	1.2 ±0.1
CH-GECO2.1	CaM	Gly21Asp Leu61Phe Ser77Thr	230	44 ±3.7	1.1 ±0.1
CH-GECO2.1	CaM	Asn109Asp	313	13 ±1.6	1.5 ±0.2
CH-GECO2.1	CaM	Ala23Asp	307	7 ±0.4	1.0 ±0.1
CH-GECO2.1	CaM	Gly21Asp Ala23Asp Ser77Thr	270	5 ±0.3	1.7 ±0.1
CH-GECO2.1	CaM	Gly21Asp Ala23Asp Leu61Phe Ser77Thr	302	23 ±1.1	1.3 ±0.1
CH-GECO2.1	CaM & FP	Gly21Asp Ala23Asp Leu61Phe Ser77Thr Asp191Gly (FP)	356	21 ±2.3	1.2 ±0.1
CH-GECO2.1	CaM & FP	Gly21Asp Ala23Asp Leu61Phe Ser77Thr Asn109Asp Asp191Gly (FP)	372	35 ±2.1	1.5 ±0.1
CH-GECO2.1	FP	Asp191Gly	211	8 ±1.4	1.0 ±0.2
CH-GECO2.1	FP	Thr147Ile	115	5 ±0.3	1.9 ±0.2
CH-GECO2.1	FP	Lys70Gln	no red fluorescent protein		
CH-GECO2.1	FP	Glu148Gln	no red fluorescent protein		
CH-GECO2.1	FP	His75Gln	158	3 ±0.3	1.4 ±0.1
CH-GECO2.1	FP	Tyr193Phe	305	5 ±0.3	1.2 ±0.1
CH-GECO2.1	CaM	Asp1Gln	307	53 ±4.2	1.7 ±0.2
CH-GECO2.1	FP	Lys166Arg	200	3 ±0.5	1.2 ±0.1

Supplementary Table 1. *Cont.*

Protein	Domain	Mutation	Response (%)	K_d (nM)	Hill Coefficient
CH-GECO2.1	FP	His172Gln	304	3 ±0.3	1.1 ±0.1
CH-GECO2.1	FP	His204Gln	285	7 ±0.4	1.3 ±0.1
CH-GECO2.1	FP	Tyr214Phe	232	8 ±0.4	1.2 ±0.1
CH-GECO2.1	FP	Trp83Phe	280	7 ±0.7	1.1 ±0.1
CH-GECO2.1	FP	Gly159Ser	184	8 ±1.0	0.9 ±0.1
CH-GECO2.1	FP	Gln163Lys	no response to Ca^{2+}		
CH-GECO2.1	FP	Gln163Met	54	2 ±0.3	1.2 ±0.2
CH-GECO2.1	FP	Gln163Asp	no response to Ca^{2+}		

Supplementary Figure 1. Absorbance scans and pH titration data for CH-GECO2.1 with R-GECO1 linkers, with or without the Gln163Lys substitution. (**A**) Absorbance spectra for CH-GECO2.1-PVV and CH-GECO2.1-PVV Q163K in the presence and absence of Ca^{2+}. In both instances there is a much larger portion of protein in the blue form and neither mutant shows a significant absorbance change in the presence of Ca^{2+}. (**B**) Absorbance spectra for CH-GECO2.1-PVV-ATA Q163K in the presence and absence of Ca^{2+}. The absorbance spectrum of CH-GECO2.1-PVV from (A) is shown for reference. (**C**) pH titration data of CH-GECO2.1-PVV and CH-GECO2.1-PVV-ATA Q163K in the presence and absence of Ca^{2+}.

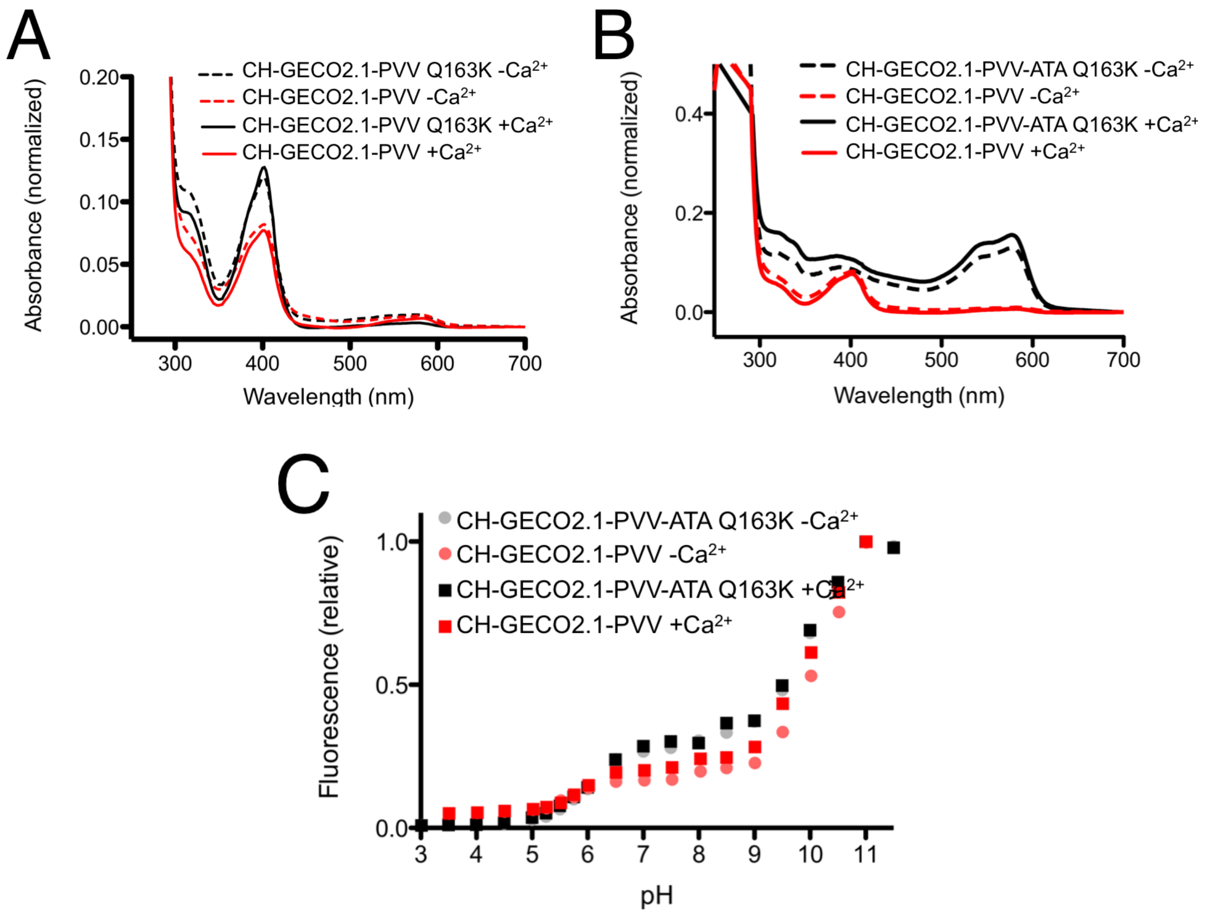

Supplementary Figure 2. The location of residues mutated in an effort to gain insight into the mechanism of CH-GECO2.1. (**A**) The hydrogen bond network connecting His75 and residues in close proximity to the chromophore, as observed in the X-ray crystal structure of mCherry (PDB ID 2H5Q) [1]. (**B**) Additional candidate residues targeted for mutagenesis, represented using the R-GECO1 crystal structure [2]. As the structure corresponds to R-GECO1, and the labels are for CH-GECO2.1, the actual side chain shown does not necessarily correspond to the amino acid name indicated in the label.

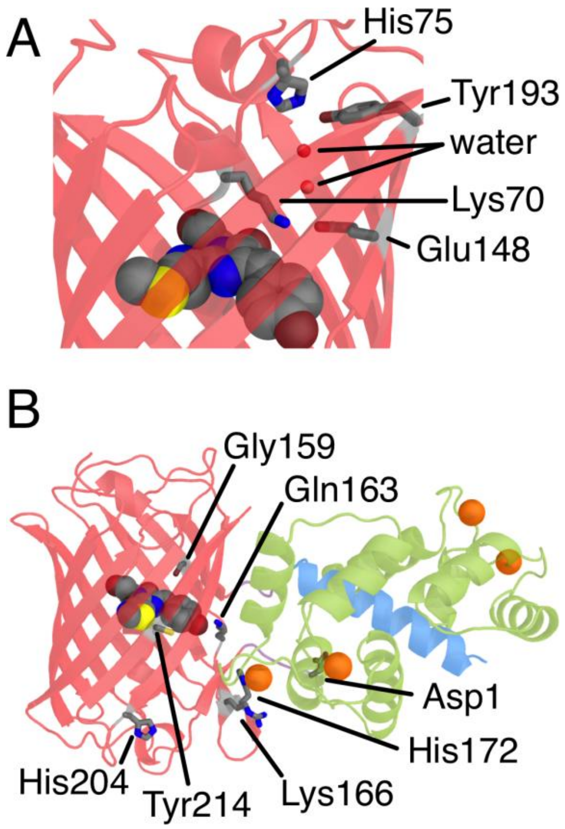

Supplementary References

1. Shu, X.; Shaner, N.C.; Yarbrough, C.A.; Tsien, R.Y.; Remington, S.J. Novel chromophores and buried charges control color in mFruits. *Biochemistry* **2006**, *45*, 9639–9647.
2. Akerboom, J.; Calderón, N.C.; Tian, L.; Wabnig, S.; Prigge, M.; Tolö, J.; Gordus, A.; Orger, M.B.; Severi, K.E.; Macklin, J.J.; *et al.* Genetically encoded calcium indicators for multi-color neural activity imaging and combination with optogenetics. *Front. Mol. Neurosci.* **2013**, *6*, 2.

© 2013 by the authors; licensee MDPI, Basel, Switzerland. This article is an open access article distributed under the terms and conditions of the Creative Commons Attribution license (http://creativecommons.org/licenses/by/3.0/).
